# Disseminated Bacillus Calmette-Guérin (BCG) infections in infants with immunodeficiency

**DOI:** 10.1186/s13104-017-2499-7

**Published:** 2017-05-05

**Authors:** Suleiman Al-Hammadi, Ahmed R. Alsuwaidi, Eman T. Alshamsi, Ghassan A. Ghatasheh, Abdul-Kader Souid

**Affiliations:** 1 Department of Pediatrics, UAE University, P.O. Box 17666, Al Ain, United Arab Emirates; 2 Department of Pediatrics, Tawam Hospital, Al Ain, United Arab Emirates

**Keywords:** Vaccines, BCG, *Mycobacterium bovis*, Danish-SSI 1331 strain, Immunodeficiency, Newborns, Tuberculosis

## Abstract

**Background:**

The Bacillus Calmette-Guérin (BCG) preparations are live-attenuated derivatives of *Mycobacterium bovis*. These products are used to vaccinate infants at birth, a practice that may result in a disseminated infection in those patients who have an unidentified immunodeficiency.

**Case presentation:**

Patients who were immunized at birth with BCG and who developed a disseminated infection are reported here to emphasize the importance of taking an extensive medical history before ‎giving the BCG vaccine. *Patient 1* has a sibling who had familial hemophagocytic lymphohistiocytosis. *Patient 2* has a severe immunodeficiency with profound lymphopenia. *Patient 3* has a sibling who had a disseminated BCG infection. *Patient 4* has two siblings with an immunodeficiency disorder; one sibling passed away in infancy and one is receiving regular immunoglobulin infusions. *Patient 5* has profound lymphopenia and his brother had cytomegalovirus (CMV) pneumonitis and passed away in infancy.

**Conclusions:**

These unfortunate events could have been avoided by compiling the relevant clinical and laboratory information. These cases also underscore the importance of a strict adherence to the BCG vaccine policies. Local and international registries that estimate the birth prevalence of primary immune deficiencies are needed prior to implementing universal BCG vaccination administration.

## Background

BCG preparations were first derived in 1921 by attenuating several *Mycobacterium bovis* (*M. bovis*) cultures. From these cultures, live vaccines were produced and distributed worldwide. However, marked genetic changes in the commercial strains have evolved over time causing the well-documented “BCG diversity” [1, 2]. In addition, strain-dependent variations in T cell activation have also been demonstrated [3, 4].

The United Arab Emirates (UAE) started to utilize the Staten Serum Institute (SSI) Copenhagen (Danish-SSI 1331) strain in 2005, which is administered to infants at birth. The vaccine must have acquired significant mutations, which may be contributing to the frequently observed adverse events in vaccinated infants. Therefore, reporting clinical experience with this vaccine is very important [5–9].

Development of firm health policies that would prevent BCG vaccine administration to vulnerable neonates is required. Five patients are described here to emphasize the importance of taking essential precautions prior to any use of this live vaccine.

## Case presentation

### Patient 1

**Fig. 1:**
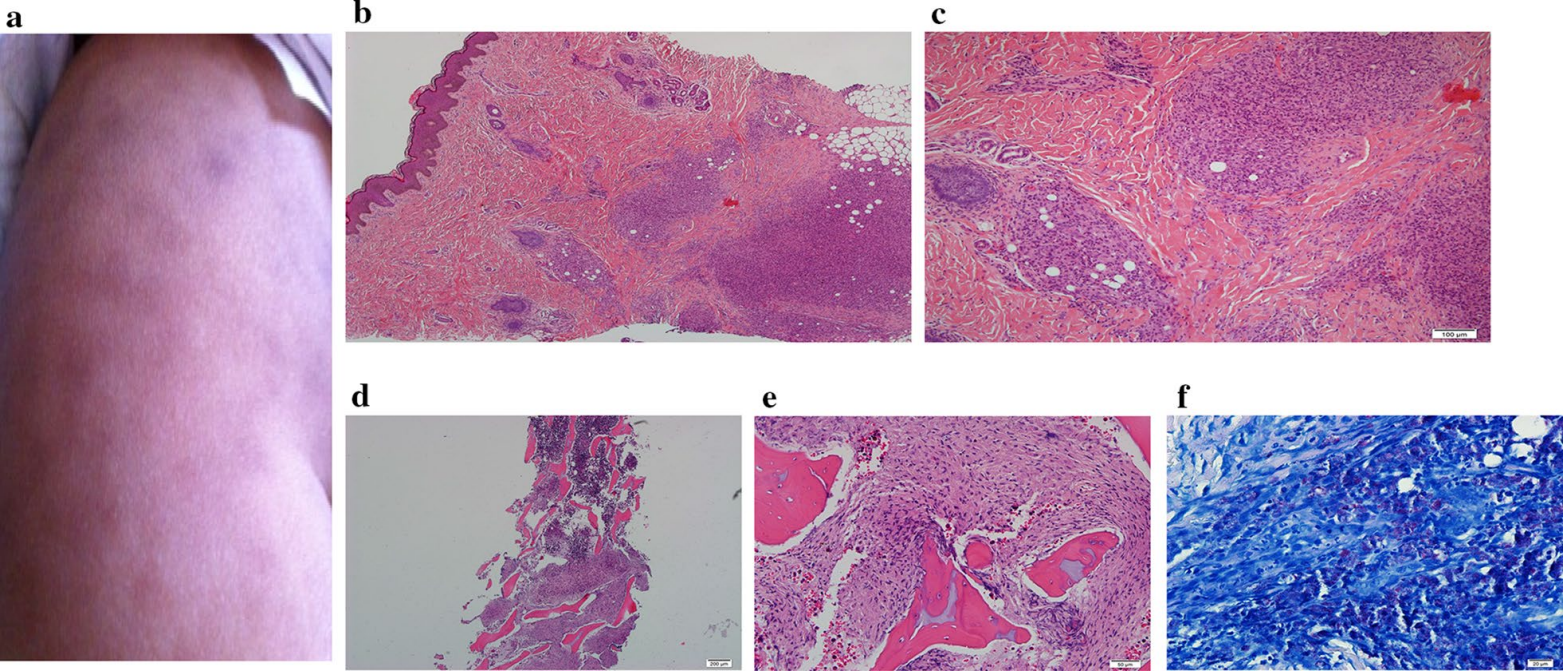
Case 1.** a** skin lesions showing erythematous nodules.** b**,** c** H&E stain of a skin excisional lesion ‎showing infiltration by histiocytes in the dermis. **d**,** e** H&E stain of marrow biopsy, showing infiltration by histiocytes. **f** marrow specimen revealing infiltration by histiocytes ingesting acid-fast bacilli

This patient received the BCG vaccine (Danish-SSI 1331) at birth. Her older brother passed away in infancy of presumed familial hemophagocytic lymphohistiocytosis (FHL). Another sibling had meningitis and died at 20 days of age. She presented at 4 months of age with persistent fever and erythematous skin nodules (Fig. 1a). Biopsies of a skin lesion (Fig. 1b, c) and bone marrow (Fig. 1d, e) revealed infiltrations by histiocytes ingesting acid-fast bacilli (AFB), Fig. 1f. Subsequently, she developed central nervous system and bone infections with mycobacterium avium-intracellulare. She was treated with rifampin, clarithromycin, ethionamide, and ethambutol.

### Patient 2

**Fig. 2:**
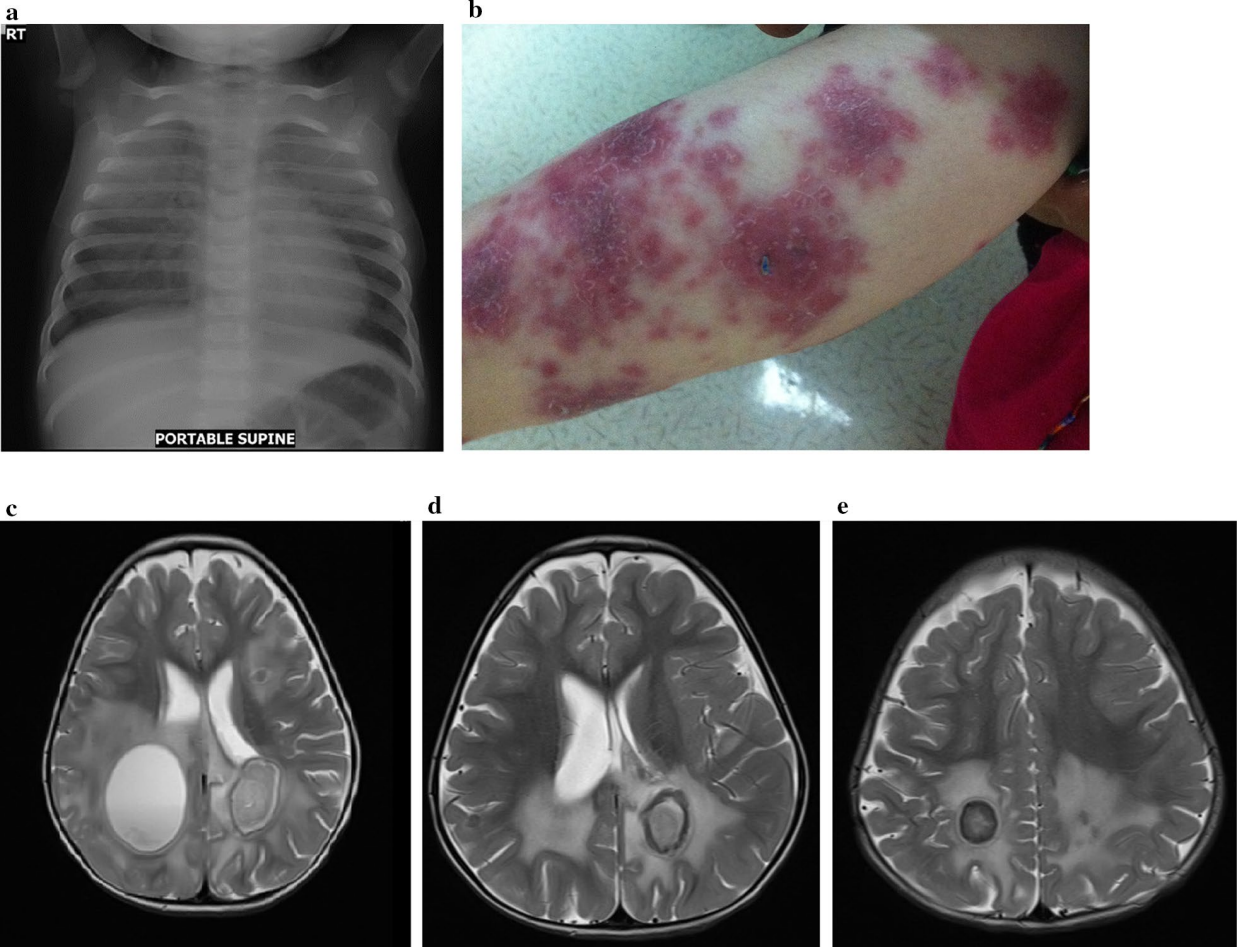
Case 2. ** a** (age 9 months): chest x-ray at her initial presentation showing bilateral pneumonia.** b** *left* forearm image showing her well-defined erythematous to brownish scaly plaques of different sizes.** c** (age 24 months): multiple cystic TB lesions with ring enhancements are evident; one in the* right* parietal lobe (measuring 48 × 45 mm and associated with edema and compression of the ipsilateral lateral ventricle) and one in the* left* parietal lobe (measuring 33 × 23 mm). Other smaller lesions are seen scattered on both cerebral hemispheres. ** d**,** e** (age 32 months): decrease in the size of these lesions as well as decrease in the thick enhancement of their peripheral walls following treatment

This patient received the BCG vaccine (Danish-SSI 1331) at birth. She presented at 9 months of age with bilateral pneumonia (Fig. 2a) and lymphopenia (lymphocyte count, 1620/µL). Subsequently, her lymphocyte counts ranged from 480 to 1060/µL, averaging 816/µL (5th percentile reference count for age = 3000/µL). At about 18 months of age, she developed skin lesions (Fig. 2b) and seizures. The brain MRI showed multiple abscesses (Fig. 2c–e). FAB smear of a drained abscess in the right parieto-occipital region revealed 3+ acid-fast bacilli; *Mycobacterium tuberculosis* complex was detected by polymerase chain reaction. The isolate was sensitive to ethambutol, rifampin, and streptomycin and resistant to pyrazinamide and isoniazid, suggestive of the *M. bovis*/BCG strain. Biopsy of a skin lesion revealed perivascular and periadnexal dermatitis; Gram stain, periodic acid–Schiff–diastase (PAS), Gomori methenamine silver (GMS), AFB, and FITE’S acid fast stains were negative. She was treated with high-dose isoniazid, ethambutol, and rifampin, and responded favorably to the treatment.

At the age of 42 months, her CD3 T-cell count was 29/µL (reference, 2110–3950/µL), CD4 T-cell count was 13/µL (920–2100/µL), CD8 T-cell count was 14/µL (810–1650/µL), CD19 B-cell count was 256/µL (420–1200/µL), and NK cell count was 1/µL. Screening for RAG1 (recombination activating gene 1), RAG2 (recombination activating gene 2), and adenosine deaminase mutations was negative. Her serum immunoglobulin levels were below the 5th percentile reference count for age. She was started on regular immunoglobulin infusions while awaiting bone marrow transplantation.

### Patient 3

**Fig. 3:**
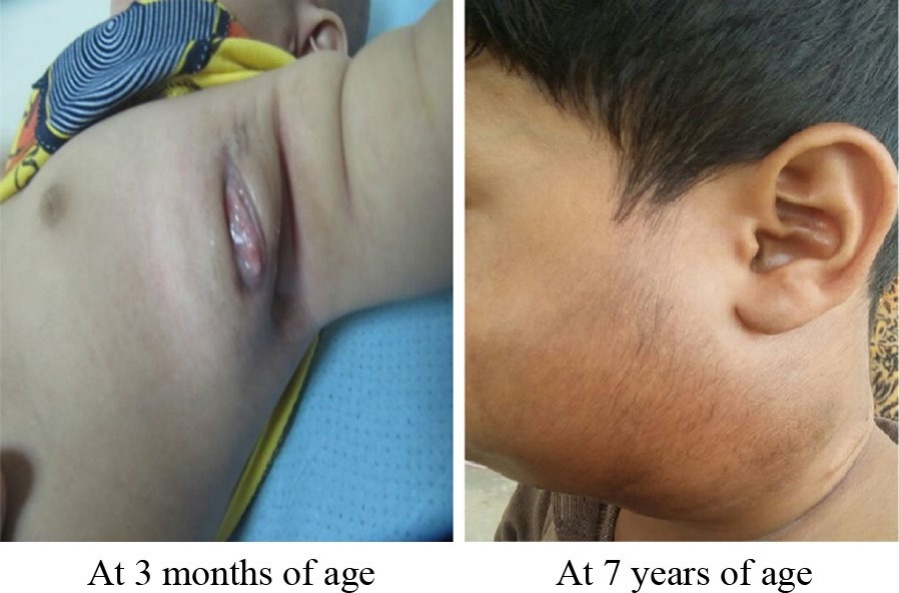
Case 3. BCG vaccine-associated chronic (recurrent) cervical lymphadenitis since birth

This patient received the BCG vaccine (Danish-SSI 1331) at birth. His older sister passed away at 6 months of age of disseminated BCG infection. He presented at 2 months of age with BCG purulent (suppurative) axillary adenitis (Fig. 3, left panel). The culture grew *Mycobacterium TB* complex. The isolate was sensitive to ethambutol, rifampin, and streptomycin and resistant to pyrazinamide and isoniazid. This susceptibility profile was suggestive of the *M. bovis*/BCG strain. He was treated with rifampin and high-dose isoniazid. At 6 months of age, he presented with enlarged cervical and inguinal lymph nodes. Ciprofloxacin and clarithromycin were added, and he responded favorably to this treatment. At 7 years of age, he continued to have chronic (recurrent) cervical adenitis (Fig. 3, right panel). His younger brother and sister were advised not to receive the BCG vaccine and they remained healthy.

### Patient 4

This patient received the BCG vaccine (Danish-SSI 1331) at birth. His older brother passed away in early infancy of overwhelming sepsis. Another 12-year-old brother is on regular immunoglobulin infusions since he was 2 years of age. The patient presented at 4 months of age with disseminated BCG infection involving axillary and cervical lymphadenitis and splenomegaly. He was treated with isoniazid and started on regular immunoglobulin infusions. At 5 months of age, his IgA and IgG levels were undetectable, and his IgM level and CD19 B-cell count were normal. These findings were suggestive of hyper-IgM syndrome, but genetic confirmation was not done. He responded favorably to the treatment.

### Patient 5

This patient received the BCG vaccine (Danish-SSI 1331) at birth. His older brother had profound lymphopenia (lymphocyte count of 220/µL at 4 months of age) and passed away in infancy of cytomegalovirus (CMV) pneumonitis. Similar to his brother, this infant had a lymphocyte count of 1100/µL at 6 days of age. Lymphocyte immunophenotyping showed: CD3 T-cell count = 703/µL (10th percentile for age = 2500/µL), CD4 T-cell count = 38/µL (10th percentile for age = 1600/µL), CD8 T-cell count = 601/µL (10th percentile for age = 560/µL), CD19 B-cell count = 59/µL (10th percentile for age = 300/µL), and NK cell count = 102/µL (normal for age). He was asymptomatic. He was treated with isoniazid and responded favorably to the treatment. He had bone marrow transplantation to treat his immunodeficiency.

## Conclusions

BCG vaccination should be avoided if any family history, clinical or laboratory evidence suggests concerns about the immune competency status of the neonate. Thus, it is essential to attain a detailed history addressing family members with probable immune deficiency or adverse effects of the vaccine prior to administering BCG; a short list of these conditions is shown in Table 1. In addition, disseminated BCG infection should be suspected in any vaccinated infants with a persistent fever or compatible illness of undetermined etiology.

**Table 1:** BCG vaccine is contraindicated in

Neonates who have
Lymphopenia, neutropenia, congenital anemia, or pancytopenia
Illness of undetermined etiology
Immunosuppressive infection (e.g., HIV or CMV)
Conceivable need for organ transplant (e.g., bone marrow transplantation)
Conceivable need for immunosuppressive therapy (e.g., steroid)
Conceivable need for cytotoxic therapy (e.g., in the presence of a tumor)
Neonates with any family members who have
Known or suspected immunodeficiency (including familial hemophagocytic lymphohistiocytosis‎)
BCG-associated complications (including suppurative adenitis)
Lymphopenia or pancytopenia due to an inherited hematologic disorder
Frequent infections or any serious health condition of unknown cause
History of death in infancy of an undetermined cause
Household where an index case of TB is suspected or confirmed

Local and international registries are necessary for estimating the birth prevalence of primary immune deficiency in the population prior to implementing universal BCG administration policies. The Canadian national surveillance program revealed a relatively high prevalence of severe combined immune deficiency among the Canadian First Nations Métis and Inuit newborns, endorsing the health authority’s policy on discontinuing the use of BCG vaccine and replacing it with enhanced TB screening and control measures [10]. Similarly, a delay in BCG administration has been suggested in order to avoid vaccinating the highly vulnerable neonates [11].

Several BCG vaccines are available worldwide. These products are prepared from *M. bovis* strains that contain diverse mutations, which create phenotypic properties that are likely to affect the adverse events of the vaccine [2]. In a randomized study involving human neonates, immune responses (*Mycobacterium*-specific polyfunctional and CD107-expressing CD4 T-cells associated with Th1 cytokines) were documented following the vaccination with various strains including the BCG-Denmark (Danish-SSI 1331) preparation [12]. It is unclear, however, whether these mounted immune responses can protect against *M. bovis* infection [13]. In one study involving white-tailed deer, the BCG Danish-SSI 1331 strain ameliorated the lymphadenopathy severity scores that followed the intratonsillar inoculation of virulent *M. bovis* [14].

General information on the BCG vaccine (indications, contraindications, monitoring, proper method of administration, and potential adverse events) are found in the product inserts. Briefly, guidelines for administering the vaccine include ensuring the correct dose (0.05 mL of the reconstituted vaccine), appropriate site of administration (deltoid region of the arm, one-third down the upper arm over the insertion of the deltoid muscle), appropriate method of injection (intradermal), and routine post-BCG assessments for early detection of adverse reactions [15]. These guidelines should be followed.

Despite high trafficking of tuberculosis (TB) in UAE [16], the prevalence of latent TB infection (LTBI) in the Emirate of Abu Dhabi among pediatric patients presenting for routine care is relatively low (0.45%) [17]. BCG vaccine may have contributed to this low prevalence in the country, especially since the prevalence of LTBI among Emirati medical students is much higher (8–10%) [18].

Prevalence of BCG adenitis varies from 0.5 to 5.8%. Medical records of 5900 neonates who received the BCG vaccine at Tawam Hospital (Al Ain City, UAE) from June 2009 to September 2010 were reviewed (unpublished observations). Thirty-five (0.6%) neonates had BCG adenitis. The median age at presentation was 3 months (range, 1.5–7 months). Lesions were on the side of the body where the injection was administered; 31 neonates had swollen axillary nodes, three had supraclavicular nodes, and one had axillary and supraclavicular nodes. Five neonates had suppuration, manifested as discharge, fluctuation, or sinus tract infections. Increased suppurative lymphadenitis caused by *M. bovis* BCG has been reported in Saudi Arabia, and the majority of the cases are related to the Danish 1331 strain [19]. The authors concluded that: “lack of nationwide data on real magnitude of BCG related adverse events warrants population centric, long term future studies” [19]. In another recent report, injection site reactions and non-suppurative adenitis were managed conservatively [20]. The outcome of suppurative adenitis was variable [20]. Therefore, suppurative adenitis should be considered a serious adverse event of the vaccine [19, 20].

In summary, health authorities should regulate BCG vaccination and enforce well-founded guidelines to avoid its administration to neonates with an immunodeficiency disorder or with a family medical history compatible with immunodeficiency. The BCG vaccine should be totally avoided if any concerns related to the immune competency status of the neonate exist. Published reports related to the clinical experience with different strains (lots) should be encouraged.

## Abbreviations

BCG: Bacillus Calmette-Guérin
*M. bovis*: *Mycobacterium bovis*
TB: tuberculosis
LTBI: latent TB infection
SSI: Staten Serum Institute
CMV: cytomegalovirus
FHL: familial hemophagocytic lymphohistiocytosis
AFB: acid-fast bacilli
